# Supplementary material for: Significant variations in tolerance to clothianidin and pirimiphos-methyl in Anopheles gambiae and Anopheles funestus populations during a dramatic malaria resurgence despite sustained indoor residual spraying in Uganda
Source: Parasit Vectors. 2025 Jun 23;18:237. doi: 10.1186/s13071-025-06867-z (PMC12183807; doi:10.1186/s13071-025-06867-z)
Supplement: Supplementary file 3 — Supplementary Material 3. [file 13071_2025_6867_MOESM3_ESM.pdf]

Supplementary material

Supplementary Table. 1 HPLC methods for different active ingredients.

	**Active Ingredient**	
	**Pirimiphos Methyl**	**Clothianidin**
**Injection Volume**	20 µL	20 µL
**Mobile Phase**	70:30 Acetonitrile: Water	93:7 Acetonitrile: Water with 0.1% phosphoric acid
**Flow Rate**	1 mL/min	1 mL/min
**Run Time**	22 min	9 min
**Wavelength**	232 nm	232 nm

Supplementary Fig. 1 Phenotypic resistance characterisation of BusiaUg and Kisumu lab strains to pyrethroids (PY) – deltamethrin and permethrin and an organochlorine (OC) – DDT, and response of wild F0 mosquitoes to clothianidin + deltamethrin (CTD+DM) and clothianidin (CTD) only.

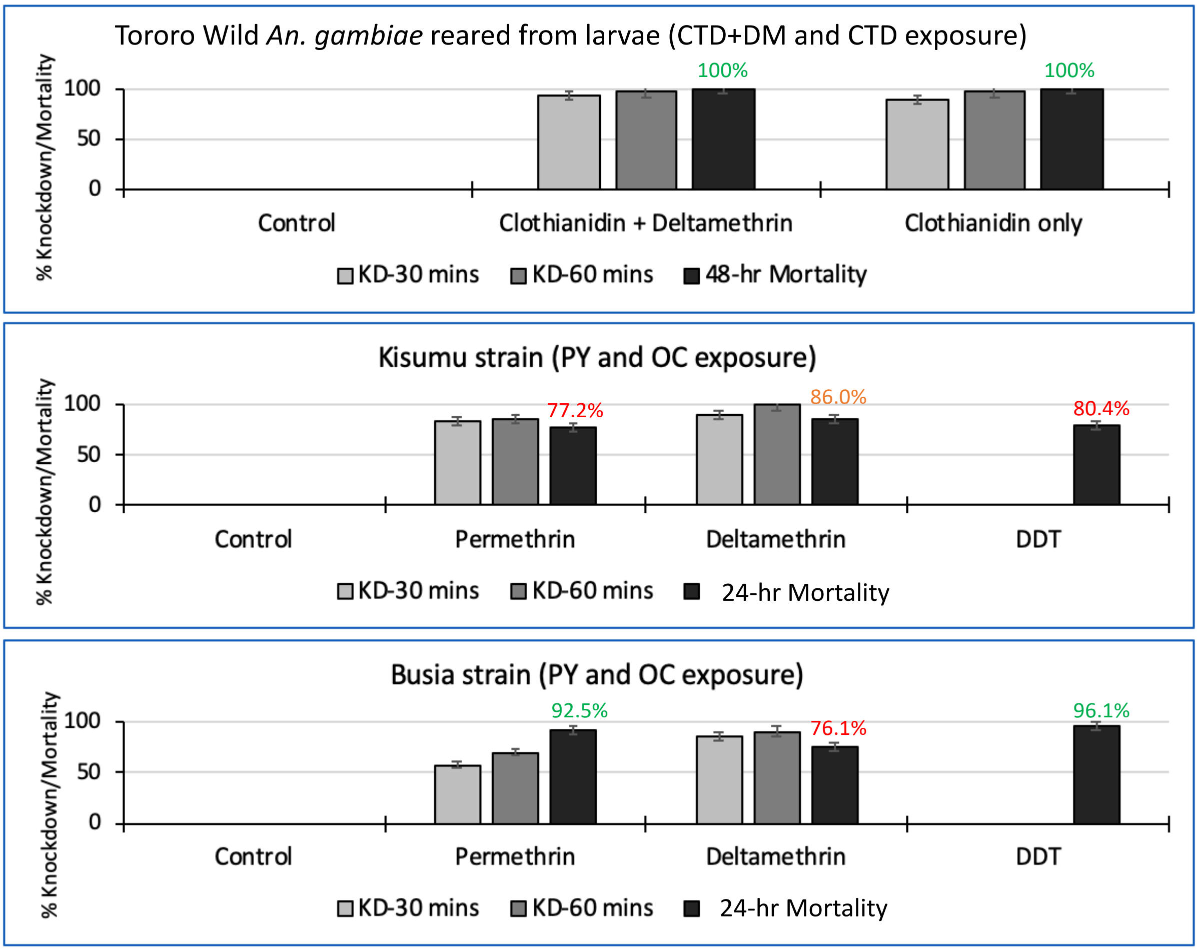

Supplementary Fig. 2 A cartoon showing the setup of cones on the walls of houses during exposure. The exposed field mosquitoes are labelled as (Ef), the exposed Kisumu strain (Ek) and the unexposed control cone (C).

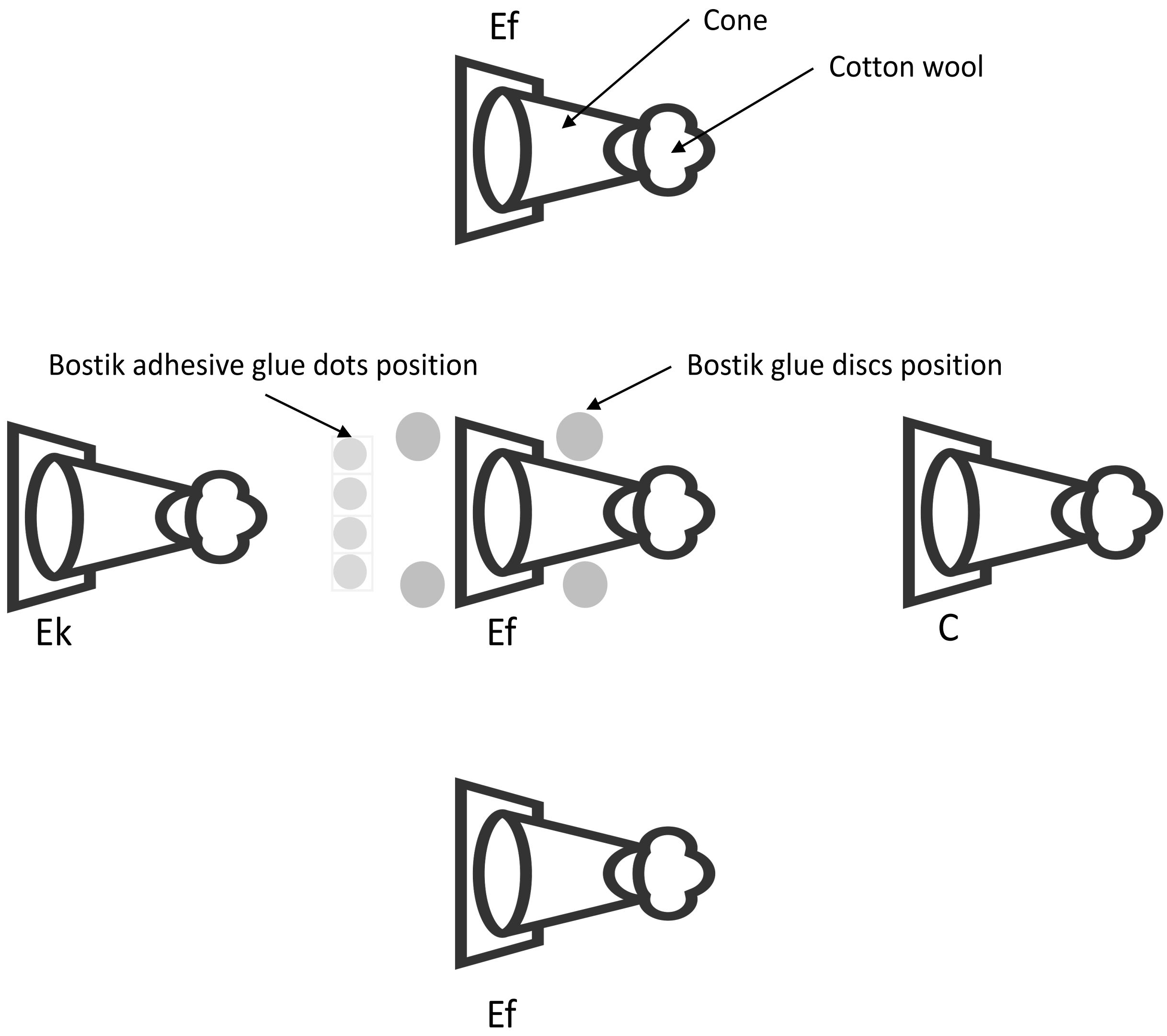

Supplementary Fig. 3 Residual concentration of insecticides sampled during spraying (0-months). A) Sumishield (clothianidin) and B) Actellic (pirimiphos-methyl).

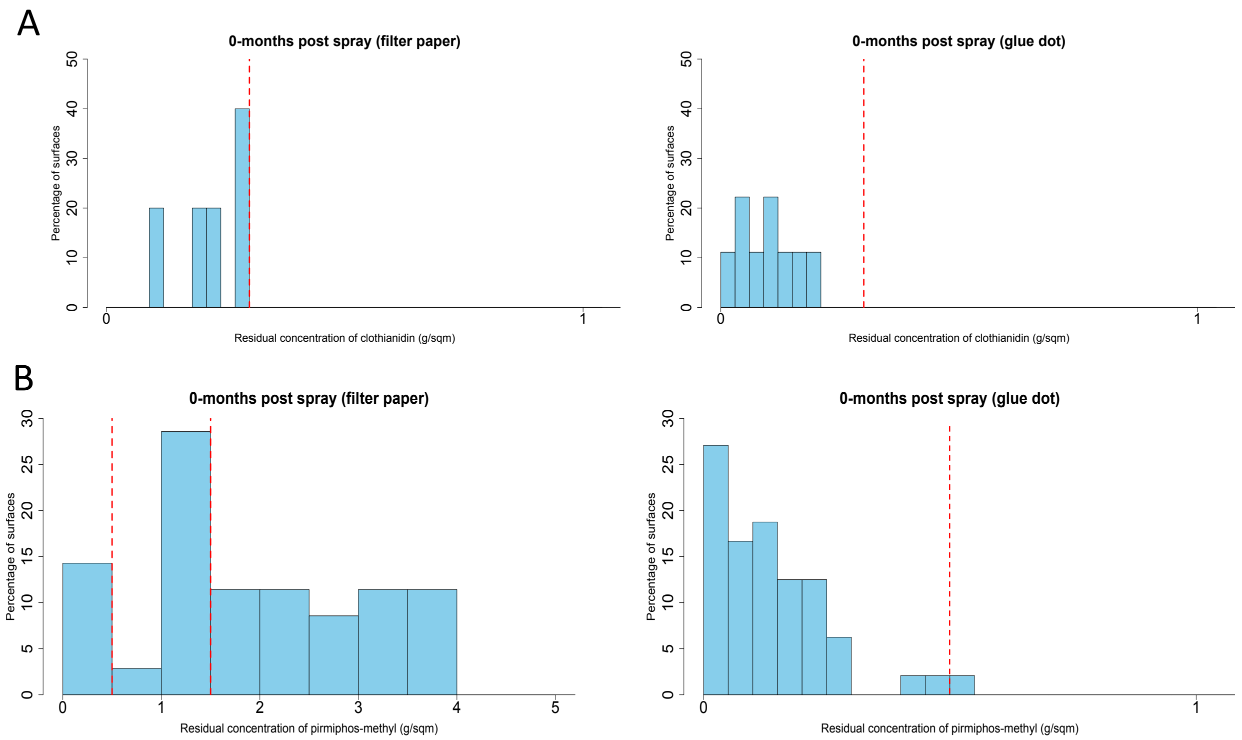

Supplementary Fig. 4 Box plot for mortality rates of exposed mosquitoes from wall cone assays in 2022 grouped by wall types (A) and part of the wall (B). Error bars represent SEM. The red dotted horizontal line is 90% mortality cut-off, below which is confirmed resistance. The level of significance is indicated by asterisks (*<0.05, **<0.01, ***<0.001) and [NS] meaning "not significant".

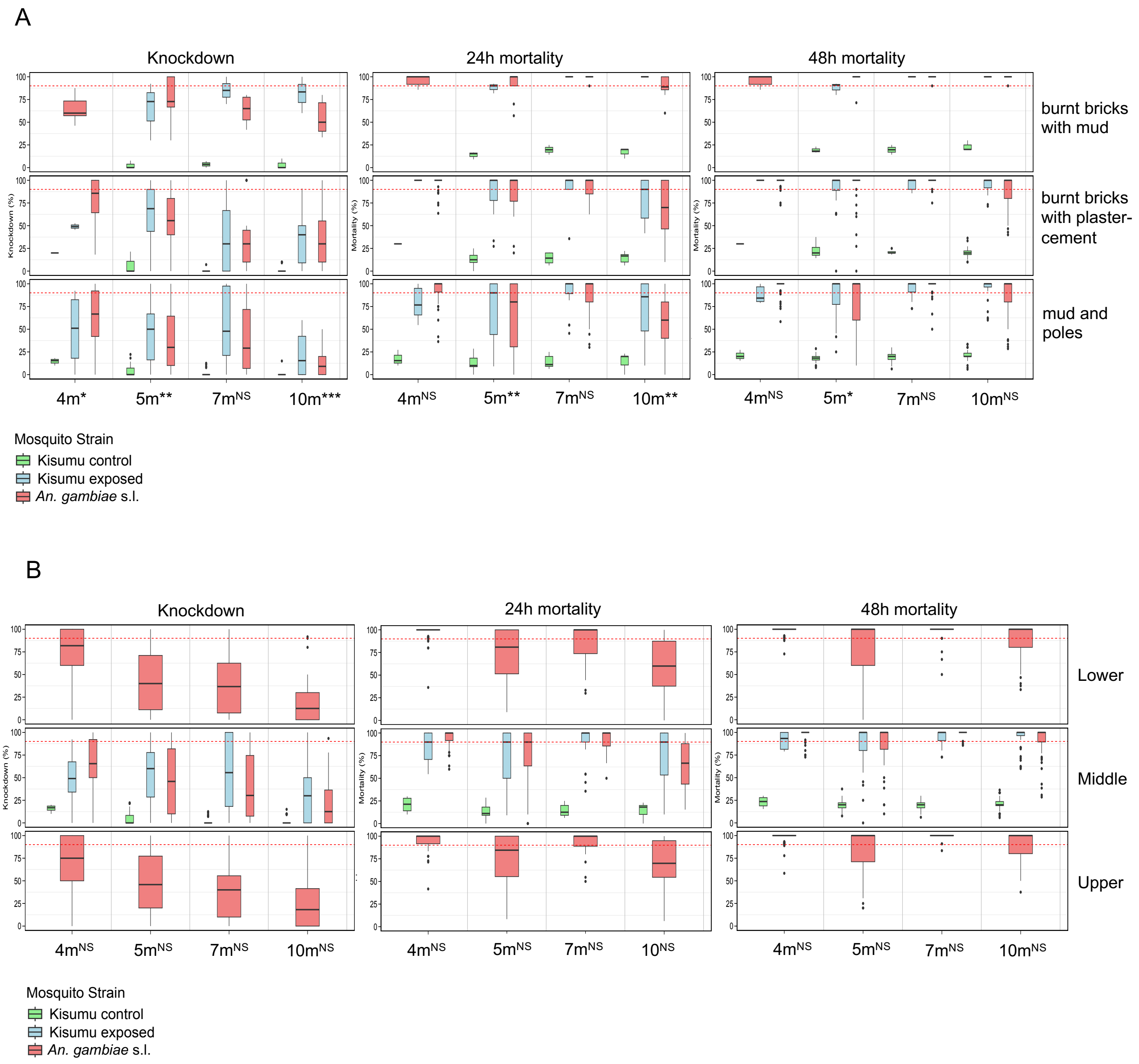

Supplementary Fig. 5 Box plot for mortality rates of only *An. gambiae* exposed mosquitoes from wall cone assays in 2023 grouped by wall types (A) and part of the wall (B). C) is the average concentration of IRS insecticides in Sumishield and Actellic houses grouped by part of the wall. Error bars represent SEM. The red dotted horizontal line is 90% mortality cut-off, below which is confirmed resistance. The level of significance is indicated by asterisks (*<0.05, **<0.01, ***<0.001) and NS meaning “not significant”.

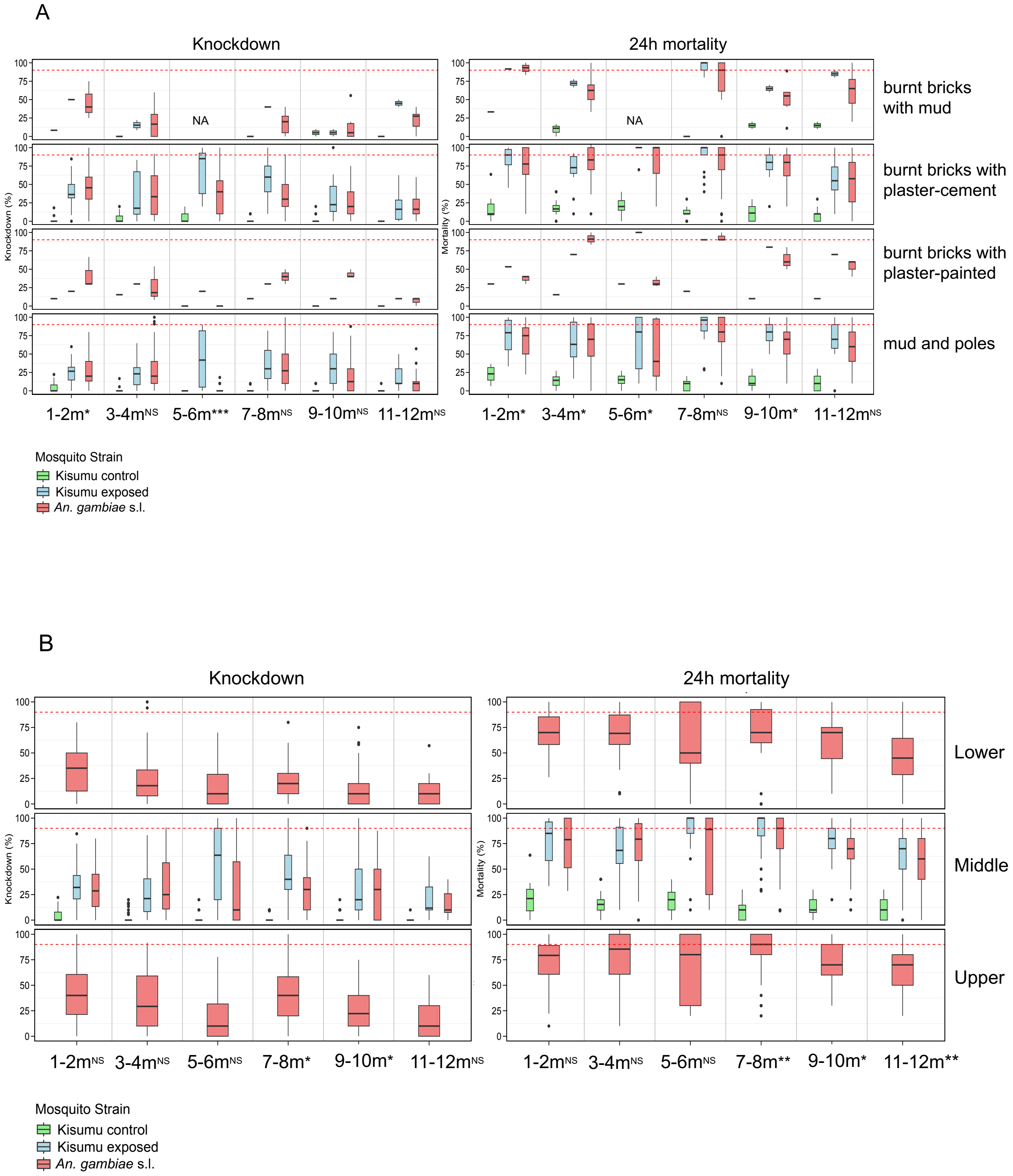

C

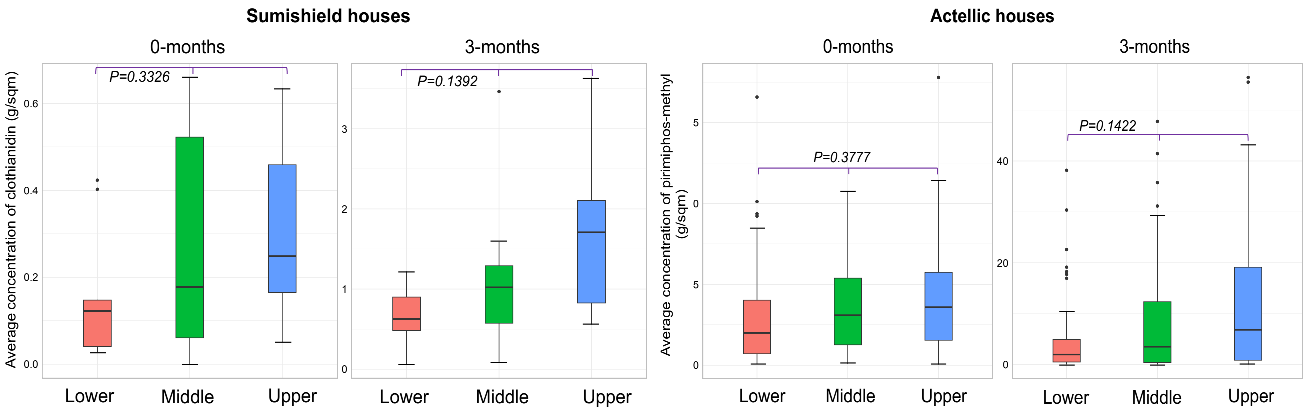
Sumishield houses
0-months
3-months
Actellic houses
0-months
3-months
Average concentration of clothianidin (g/sqm)
Average concentration of pirimiphos-methyl (g/sqm)
P=0.3326
P=0.1392
P=0.3777
P=0.1422
Lower
Middle
Upper
Lower
Middle
Upper
Lower
Middle
Upper
Lower
Middle
Upper

Supplementary Fig. 6 Bar plot for mortality rates of only wild *An. gambiae* mosquitoes exposed to non-sprayed walls in 2022 and 2023 with a table showing the average concentration of clothianidin detected in those houses.

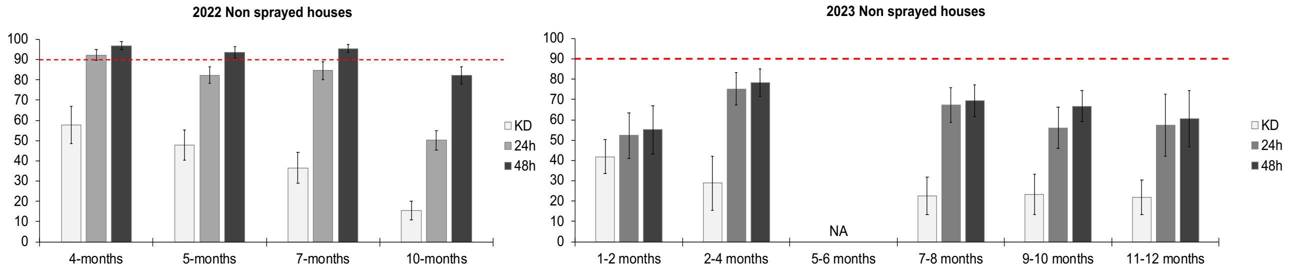

	2022 (n=9)	2023 (n=2)
Average clothianidin concentration (g/sqm)	0.0184	0.4237
Average pirimiphos-methyl concentration (g/sqm)	NA	NA
